# Which Is the Most Appropriate PI3K Inhibitor for Breast Cancer Patients with or without PIK3CA Status Mutant? A Systematic Review and Network Meta-Analysis

**DOI:** 10.1155/2020/7451576

**Published:** 2020-12-03

**Authors:** Shu Wang, Mingyue Liu, Siheng Lian, Naiming Liu, Guibin Zhang, Qingchun Zhao, Yingshi Zhang, Lingyan Jian

**Affiliations:** ^1^Department of Pharmacy, Shengjing Hospital of China Medical University, Shenyang 110004, China; ^2^Department of Clinical Pharmacy, Shenyang Pharmaceutical University, Shenyang 110016, China

## Abstract

**Objective:**

The phosphatidylinositol 3-kinase (PI3K) signaling pathway is a promising treatment target for patients with breast cancer (BC). Our study aimed to evaluate the most effective and safe PI3K inhibitor for patients with BC, especially in PIK3CA mutation.

**Methods:**

Electronics databases were systematically searched from their inception to June 2020 for published randomized controlled trials (RCTs) comparing PI3K inhibitor therapy versus non-PI3K inhibitor therapy in patients with BC that mentioned or reported data of PIK3CA-mutated patient subgroups. Eligible RCTs had to report at least one of the following clinical outcomes: objective response rate (ORR), progression-free survival (PFS), or adverse events (AE).

**Results:**

Nine eligible RCTs involving 3872 BC patients and four PI3K inhibitor therapy arms (i.e., alpelisib, buparlisib, pictilisib, and taselisib) were included. In evaluating ORR, beneficial significant results of PI3K inhibitors could be found in the PIK3CA mutated group (1.952, 1.012 to 3.766); analogous results could also be found in 6m-PFS (1.519, 1.144 to 2.018) and PFS from HR data (-0.346, -0.525 to -0.168). From pairwise and network meta-analyses, buparlisib showed the most favorable ORR, as it was significantly different from fulvestrant in the PIK3CA-mutated patient group (2.80, 1.56 to 5.03). Alpelisib ranked first in the assessment of 6m-PFS and was significantly different from fulvestrant in the PIK3CA-mutated group (2.33, 1.45 to 3.44). The above PI3K inhibitors had good safety with few serious AEs. PROSPERO registration CRD42020193932.

**Conclusion:**

The PI3K inhibitors alpelisib and buparlisib appear to have superior efficacy and safety therapeutic choices for patients with BC, especially in PIK3CA-mutated patients.

## 1. Introduction

Breast cancer (BC) is the most common malignancy among women worldwide and is the second leading cause of tumor-related death [1, 2]. Metastasis occurs in approximately 30% of women who are diagnosed with BC because of chemotherapy resistance [3]. Moreover, metastatic tumors are less sensitive to chemotherapy, and when the patient enters the advanced stage, their prognosis is worse [4]. Therefore, novel therapeutic strategies for novel targets to delay BC progression are determined in this setting.

The activation of phosphatidylinositol 3-kinase (PI3K) signaling pathway plays a crucial role in cell growth, autophagy, metabolism, and cell survival [5, 6]. The PIK3CA (encoding p110*α*, the catalytic subunit of PI3K*α*) mutant may contribute to treatment resistance in BC, which activates the oncogene signaling of PI3K/AKT. PIK3CA is mutated in approximately 35% of all breast cancers and is more frequent in ER-positive BC [7]. In addition, BC with PIK3CA mutations is less responsive to chemotherapy and anti-HER2 therapy [8]. Thus, the PI3K signaling pathway is a promising treatment target for patients with BC, especially in PIK3CA-mutated patients.

There are currently four types of PI3K inhibitors: pan-PI3K inhibitors, including buparlisib (BKM120) and pictilisib (GDC-0941), and isoform-specific PI3K inhibitors, including alpelisib (BYL719) and taselisib (GDC-0032), which have all been shown to be efficacious and safe in published studies [9, 10]. However, it is unclear which PI3K inhibitors are the most suitable for patients; this issue is important for patients with different receptor types. No previous systematic reviews have provided the most suitable treatment strategies with meta-regression and network meta-analysis.

## 2. Methods

This systematic review and network meta-analysis was performed in accordance with the extension Preferred Reporting Items for Systematic Reviews and Meta-analyses (PRISMA) guidelines [11]. This research protocol was registered with the PROSPERO registry (CRD42020193932) [12].

### 2.1. Search Strategy and Study Selection

The PubMed, Embase, and Cochrane Library electronic databases were searched in all languages from inception until June 2020 using keywords (i.e., PI3K, PIK3CA mutate, breast cancer) and their MeSH terms (see detailed characteristics in Table S1). The reference lists of the relevant publications were also searched to identify additional eligible articles. Randomized controlled trials (RCTs) that compared the efficacy and safety of the PI3K inhibitor group versus the non-PI3K inhibitor group were considered, and subgroups of the population with PIK3CA mutations had to be mentioned for a study to be eligible. Menopause status and receptor status (positive or not positive) were used as indicators of restriction. Reviews, dose-dependent studies, basic original studies, and single-arm studies were excluded. Moreover, duplication data studies and studies without useful data were excluded.

### 2.2. Data Extraction, Outcomes, and Risk of Bias Assessment

After systematically screening studies, the following data were extracted and entered into a prestructured form: first author, publication year, study type, sample size, gender, age, cancer type, population, ECOG score, cancer stage, PI3K inhibitor regimen, intervention arm, control arm, and treatment period. In addition, the primary efficacy outcome was the overall response rate (ORR). Local BC response was measured according to the modified criteria for response evaluation in solid tumors (mRECIST). mRECIST defines the overall response into four main categories: complete response (CR), partial response (PR), progressive disease (PD), and stable disease (SD). However, the ORR, including CR and PR, and treatment continued until disease progression [13]. The secondary efficacy outcome was progression-free survival, which was defined as the time from randomization to either first disease progression or death. Safety outcomes included adverse effects (AE) of gastrointestinal disorders, general and skin disorders, metabolism, and nervous system disorders.

The risk of bias of the individual studies was assessed using the Cochrane risk of bias tool [14]. Random sequence, allocation concealment, blinding of participants and personnel, blinding of outcome assessment, incomplete outcome data, selective reporting, and other bias were assessed to determine the risk of bias. All RCTs were classified as low risk, high risk, or unclear risk of bias. We also estimated the certainty of evidence for each direct comparison according to the GRADE framework for pairwise meta-analysis [15]. Study selection, risk of bias assessment, and evidence quality evaluation were independently conducted by two investigators (WS and LMY), and any discrepancies were resolved by consensus and arbitration by a panel of adjudicators within the review team (ZYS and ZQC).

### 2.3. Data Synthesis and Statistical Analysis

In our systematic review and network meta-analysis, we mainly considered the ORR, PFS, and AE of the intervention group with the PI3K inhibitor versus the control group with the non-PI3K inhibitor. We used subgroup meta-analysis and meta-regression from PIK3CA mutation status and PI3K inhibitor type to determine the most suitable PI3K inhibitor type in PIK3CA-mutated patients. For our included pairwise meta-analysis outcomes, dichotomous data were summarized by odds ratios (ORs) with their 95% confidence intervals (CIs) and hazard ratios (HRs) with their 95% CIs from survival data. Heterogeneity was considered to be present when the *I*^2^ statistic was greater than 50% or the *p* value was less than 0.05, and random effects models were utilized to assess the accuracy regardless of the results of the heterogeneity test [16]. In addition, a *p* value less than 0.05 from the meta-regression was used to determine the source of heterogeneity [16]. Moreover, in Begg's test and Egger's test, a *p* value less than 0.05 indicated the presence of publication bias in the pairwise meta-analysis.

Additionally, we performed a network meta-analysis to further determine which of the PI3K inhibitors (alpelisib, buparlisib, taselisib, and pictilisib) were most effective for a given population (PIK3CA-mutated population or total patients). Then, ORs and corresponding 95% credible intervals (CrI) were obtained from the random effects model. The surface under the cumulative ranking curve (SUCRA) values produced a network meta-analysis to obtain the front ranking interventions [17], which ranged from 0 to 1, with a higher SUCRA score indicating that the intervention has a high likelihood of providing the best therapeutic effect. Inconsistencies between sources of evidence were statistically assessed globally and locally [18] when a direct connection between two treatment arms was not available, and the results were based on indirect evidence. As a result, all interventions were assumed to be coherent. We produced comparison-adjusted funnel plots to explore publication bias in the network meta-analysis. All the aforementioned pairwise and network meta-analyses were conducted with StataMP version 14.0.

## 3. Results

### 3.1. Systematic Review and Characteristics

**Figure 1:**
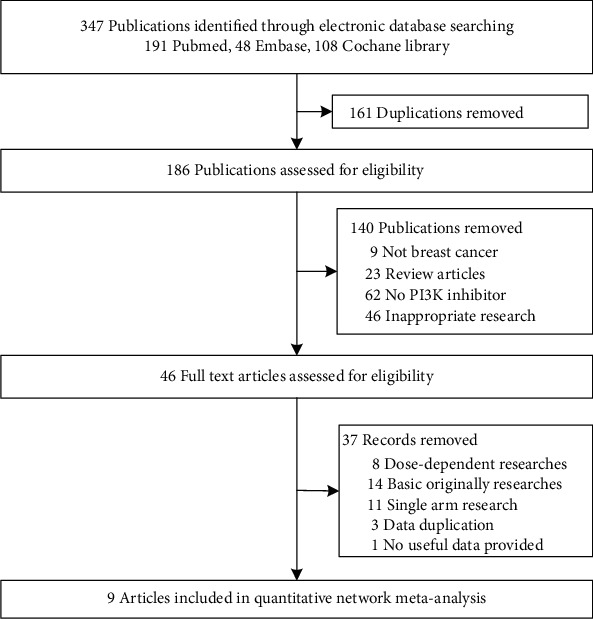
Flowchart of RCT selection.

We identified 168 publications from initial electronic databases. After removing duplicates and screening titles and abstracts, 46 full-text reports were retrieved and reviewed. Immediately after removing articles that could not provide valid data, only nine RCTs were included in the systematic review and network meta-analysis (Figure 1) [19–27]. A total of 3872 patients were enrolled to receive four different PI3K inhibitor interventions with treatment by alpelisib, buparlisib, taselisib, and pictilisib in patients with subgroups of PIK3CA-mutated patients or mentioned a part of the mutation-related population.  Table 1 presents the baseline summarized characteristics of the intervention PI3K inhibitor type, control type, receptor type, and population of every included study, and the baseline was balanced in the enrolled RCTs (see detailed characteristics in Table S2). All included RCTs had acceptable quality, with 6 of high quality and 3 of unclear quality (Figure S1).

### 3.2. Pairwise Meta-Analysis for Efficacy in BC

**Figure 2:**
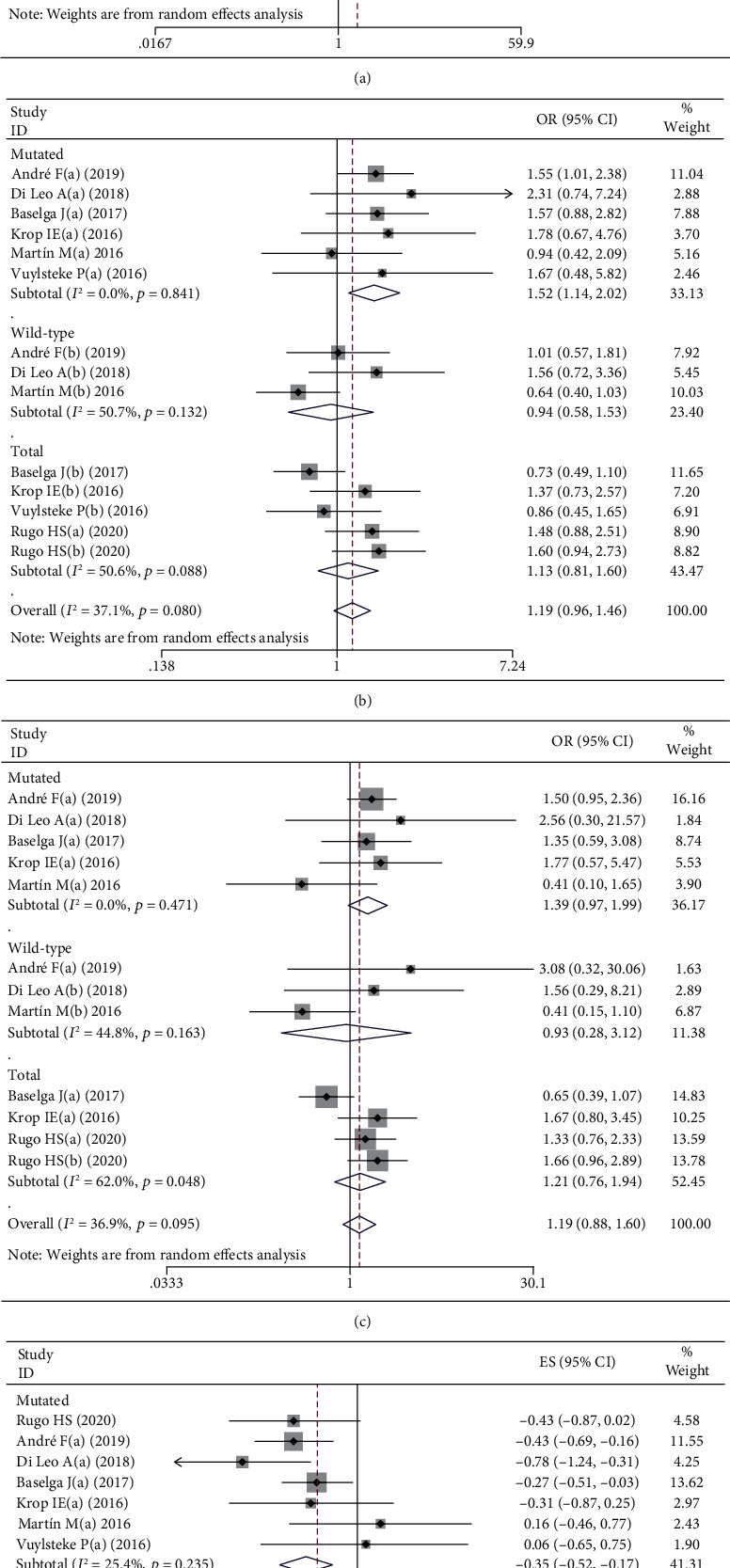
Forest plot for intervention with PI3K inhibitor versus control for objective response rate (a), 6-month progression-free survival (b), 1 year progression-free survival (c), and hazard ratio for progression-free survival (d) in the PIK3CA-mutated subgroup and the PIK3CA wild-type subgroup patients with breast cancer.

Seven of our included nine RCTs provided ORR efficacy data, and significant differences were found, indicating that the application of PI3K inhibitors may benefit overall BC patients (OR = 1.539, 95% CI: 1.074 to 2.204); there was substantial heterogeneity in this outcome (*p* = 0.015, *I*^2^ = 54.6%). In the subgroup meta-analysis by PIK3CA-mutated status, a significant outcome could only be found in the PIK3CA-mutated subgroup (1.952, 1.012 to 3.766); there was substantial heterogeneity (0.030, 62.6%) and a moderate certainty of evidence. Moreover, we conducted a further subgroup analysis to observe whether there are differences in efficacy between different PI3K inhibitors. We noticed that in the subgroups of alpelisib (2.474, 1.410 to 4.343) and taselisib (2.093, 1.094 to 4.002), significant differences could be found. However, the results are based on only one original study, and the certainty of evidence is low. Generally, no significant differences were found according to the metaregression (*p* = 0.306, 0.785). Due to the existence of heterogeneity and publication bias, a low to moderate certainty of evidence was determined, and the sensitivity analysis confirmed that the results were not affected (Figure S3A). We only found that PI3K inhibitors can improve the ORR, especially in PIK3CA mutant patients (Table 2, Figure 2(a)).

For the outcomes of PFS, first, we considered the 6m-PFS [1, 2, 4, 5, 7–9], and the subgrouping method was as described above. We found that significant differences could only be observed in the subgroup of PIK3CA patients (1.519, 1.144 to 2.018), with no heterogeneity (0.841, 0.0%), and the subsubgroup of alpelisib. However, in the buparlisib group, there was evidence that its application may improve 6m-PFS, with no heterogeneity (1.427, 0.924 to 2.205; 0.396, 0.0%). In summary, no significant source of heterogeneity was found from the meta-regression; publication bias was found in the overall group; most of our outcomes had acceptable certainty of evidence; and the sensitivity analysis did not affect the outcomes (Table 2, Figure 2(b), Figure S3B).

In addition, we also took 1y-PFS, 1.5y-PFS, and 2y-PFS into consideration. No significant differences were found in the overall and subgroup meta-analyses for 1y-PFS, and a significant effect could be found in the PIK3CA-mutated group with no heterogeneity (1.392, 0.972 to 1.992; 0.471, 0.0%; Figure 2(c)). In evaluating 1.5y-PFS, significant results could be found in the overall group, with no heterogeneity (1.506, 1.071 to 2.119, 0.892, 0.0%), and a significant effect could also be found in the PIK3CA-mutated group (1.577, 0.907 to 2.740; 0.865, 0.0%). For 2y-PFS, no significant differences were found in overall or all subgroups. For the above indicators, the source of heterogeneity could not be detected by meta-regression, and publication bias was often discovered in the overall results, with an acceptable certainty of evidence (Table 2).

For the PFS outcomes from HR data, significant differences were found in the overall group (-0.271, -0.369 to -0.173), PIK3CA-mutated subgroup (-0.346, -0.525 to -0.168), and mutated and wild-type total subgroup (-0.238, -0.364 to -0.112) with low heterogeneity. In addition, for the sub-subgroup meta-analysis in PIK3CA-mutated patients, significant differences could be found in the PI3K inhibitors of alpelisib (-0.431, -0.658 to -0.203) and buparlisib (-0.324, -0.526 to -0.123) with substantial heterogeneity. In the above outcomes, meta-regression could not determine the source of heterogeneity, and there was low-to-high certainty of evidence due to publication bias (Table 2, Figure 2(d)).

Generally, the application of PI3K inhibitors may benefit BC patients, especially PIK3CA-mutated patients. Moreover, compared with taselisib and pictilisib, alpelisib and buparlisib may be more effective. However, for the PIK3CA mutated and wild-type total subgroups, whether a PI3K inhibitor is beneficial and which one is the most effective PI3K inhibitor can only be determined by performing a network meta-analysis.

### 3.3. Network Meta-Analysis for Efficacy in BC

**Figure 3:**
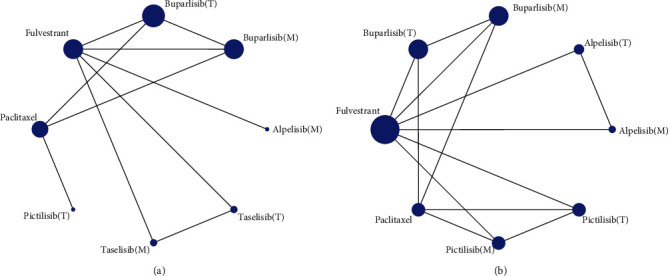
Network plot for all interventions for objective response rate (a) and 6 months progression-free survival (b) in the PIK3CA-mutated subgroup and the PIK3CA-mutated and wild-type total subgroup patients with breast cancer.

**Figure 4:**
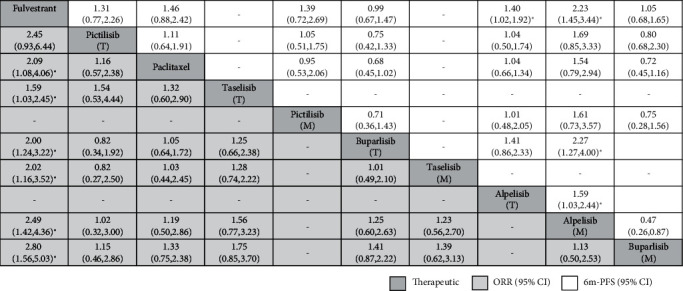
Summary intervention effects from network meta-analysis for objective response rate and 6 months progression-free survival in the PIK3CA-mutated subgroup and the PIK3CA-mutated and wild-type total subgroup patients with breast cancer according to SUCRA score.

Network meta-analysis included all interventions for ORR (Figure 3(a)), and all interventions for 6m-PFS (Figure 3(b)) were presented as network plots in the PIK3CA-mutated subgroup and the PIK3CA-mutated and wild-type total subgroup patients with breast cancer. In terms of ORR, fulvestrant ranked the lowest. Compared with fulvestrant, the application of buparlisib in PIK3CA-mutated patients ranked first, with significant differences (2.80, 95% CrI: 1.56 to 5.03), followed by alpelisib in PIK3CA-mutated patients (2.49, 1.42 to 4.36), taselisib in PIK3CA-mutated patients (2.02, 1.16 to 3.52), buparlisib in the total population (2.00, 1.24 to 3.22), taselisib in the total population (1.59, 1.03 to 2.45), paclitaxel (2.09, 1.08 to 4.06), and pictilisib in the total population (Figure 4).

In terms of 6m-PFS, compared with fulvestrant, alpelisib in PIK3CA-mutated patients (2.23, 1.45 to 3.44) ranked first, followed by ALPELISIB in the total population (1.40, 1.02 to 1.92), buparlisib in PIK3CA-mutated patients, pictilisib in PIK3CA-mutated patients, buparlisib in the total population, and paclitaxel and pictilisib in the total population. Significant differences could also be found in the intervention of alpelisib in PIK3CA-mutated patients versus buparlisib in PIK3CA-mutated patients (2.13, 1.15 to 3.85), alpelisib in the total population (1.59, 1.03 to 2.44), and buparlisib in the total population (2.27, 1.27 to 4.00; Figure 4). The publication bias for ORR was high as shown in the comparison-adjusted funnel plots (Figure S2). Therefore, for patients with BC, especially for patients with PIK3CA mutations, the PI3K inhibitors alpelisib and buparlisib are the most effective.

### 3.4. Pairwise Meta-Analysis for Safety in BC

For the safety outcomes of PI3K inhibitors, we only categorized the included RCTs based on different PI3K inhibitors, and we did not categorize the population based on PIK3CA-mutated status (Table 3). For gastrointestinal disorders, all kinds of PI3K inhibitors may increase the risk of diarrhea, especially grade 3-5 diarrhea. For nausea, all types of PI3K inhibitors may increase the risk of nausea. However, it did not increase the risk of grade 3-5 nausea. In terms of vomiting, only alpelisib significantly increased the incident risk of vomiting. In terms of decreased appetite, all PI3K inhibitors, including PI3K inhibitors, may increase the risk of decreased appetite in both all grades of AEs and grade 3-5 AEs. In the assessment of stomatitis, including the PI3K inhibitors may increase stomatitis for all grades, and a significant difference could only be found in the buparlisib group of grade 3-5 AEs.

For general and skin disorders, in consideration of fatigue, all PI3K inhibitors could increase the risk of all grade fatigue. For 3-5 grades of AE, a significant difference could also be found overall and in the buparlisib group. In terms of rash, the application of alpelisib and buparlisib may increase the AEs, while similar results could also be found in grade 3-5 AEs. For metabolism and nervous system disorders, alpelisib and buparlisib could increase the incident risk of hyperglycemia in all grades and grade 3-5 AEs. For headache, none of our included PI3K inhibitors could significantly increase the incident risk of all grades and grade 3-5 AEs. In summary, PI3K inhibitors may increase the risk of gastrointestinal disorders, general and skin disorders, and metabolism and nervous system disorders. However, the incidence of AEs was similar among several PI3K inhibitors (Table 3).

## 4. Discussion

In this systematic review and network meta-analysis, we comprehensively summarize the comparative efficacy and safety of PI3K inhibitor (alpelisib, buparlisib, taselisib, and pictilisib) treatments for patients with any stage and any receptor type of BC. First, the efficacy outcomes of ORR and PFS were evaluated by pairwise meta-analysis. Compared with non-PI3K inhibitor therapy, PI3K inhibitors could increase efficacy in the overall population, especially in patients with PIK3CA mutations. Second, from the sub-subgroup meta-analysis and network meta-analysis for ORR and 6m-PFS, alpelisib and buparlisib have the best therapeutic effect, especially in BC patients with PIK3CA mutations. Third, PI3K inhibitors may increase the incidence risk of gastrointestinal disorders, general and skin disorders, metabolism, and nervous system disorders. However, there was no significant difference among the four inhibitors. Therefore, for BC patients with PIK3CA mutations, if there is no intolerable AE, PI3K inhibitors are recommended for therapy, especially alpelisib and buparlisib. If the patients have not been tested for PIK3CA gene mutations, a PI3K inhibitor is also recommended due to its curative effect.

Compared with the reported meta-analyses focusing on PI3K inhibitor treatments for patients with BC mutated PIK3CA [10], our present network meta-analysis had several strengths. First, our study not only analyzed the effectiveness of PI3K inhibitors but also analyzed their safety. To be effective on the basis of safety is the result we want to accept; many published high-quality studies have also proven that PI3K inhibitors have acceptable safety and good tolerance [28, 29]. Second, we noticed that PI3K inhibitors are indeed more effective in patients with PIK3CA mutations, mainly because the PI3K/AKT pathway is activated through PIK3CA or AKT1 mutations and PTEN loss in BC [30, 31], which also proves that the PI3K intracellular signaling pathway plays an important role in BC [32]. For all patients with BC who do not distinguish the PIK3CA mutation type, because of the relatively expensive price of genotype detection, a PI3K inhibitor is recommended as a therapeutic strategy, which may produce a curative effect. Third, from our pairwise and network meta-analysis, we found that the most effective PI3K inhibitors may be alpelisib and buparlisib. Analogous results have been obtained in some published studies [33, 34], and the safety of the above two agents could also be tolerated.

In the original studies we have included, most of the patients in the study are postmenopausal patients with BC, so the comparison of the study is mostly combination therapy of PI3K inhibitor+fulvestrant vs. fulvestrant group alone, and the majority of the patients are HR-positive and HER2-negative. However, our meta-analysis did not limit the receptor types of patients with BC. Some studies pointed out that activating PIK3CA mutations have been linked to the development of resistance to HER2-targeted agents, and the chemotherapy-free regimen of buparlisib plus trastuzumab also demonstrated an acceptable safety profile [35]. There were also some studies indicated that PI3K inhibition and endocrine therapy have synergistic effects in HR+ patients with BC, particularly in tumors with biological indicators of pathway activation, such as PIK3CA mutations [36, 37]. Buparlisib and pictilisib are pan-PI3K inhibitors, and this broad inhibition may potentially lead to a higher risk of AE. Specific PI3K inhibitors, including alpelisib and taselisib, and the specificity of alpelisib against the p110a catalytic isoform provided additional efficacy and a better toxicity profile [9]. Based on our research results and the above literature studies, we believe that HR-positive and/or HER2-positive breast cancer patients could benefit from treatment with PI3K inhibitors (alpelisib and buparlisib), especially in postmenopausal women with endocrine therapy, and the specific mechanism needs to be further researched.

There are also some limitations among our network meta-analysis. First, the sample size was small, with only 3872 patients, and only 9 articles were included in our network meta-analysis. Four PI3K inhibitors were included in 9 studies, and some agents had only one available research report, so our results may be biased. Second, our efficacy results only reported ORR and PFS but did not report the overall survival rate (OS) data. This is probably because the clinical trials we included are in the ongoing stage, and the OS data have not been obtained. Only one study determined that OS results were in favor of buparlisib+fulvestrant versus placebo+fulvestrant, proving that patients benefited from PI3K inhibitors in the long term [38]. Third, publication bias was frequently found in the overall outcomes of efficacy results. However, publication bias was lower in the subgroup meta-analysis, which proves that our method of subgroup analysis was reasonable and appropriate.

In conclusion, from this network meta-analysis, PI3K inhibitors of alpelisib and buparlisib seem to have superior efficacy and safety treatment choices for patients with BC. The application of the PI3K inhibitor may be beneficial to all subjects. Further studies, such as prespecified RCTs of patients treated with PI3K inhibitors of alpelisib and buparlisib, are required to be more comprehensive and similar and reported separately according to different receptor types of patients with BC to determine the most appropriate PI3K inhibitors for the most suitable patients.

## Figures and Tables

**Table 1:** Baseline characteristic of included RCTs.

Intervention PI3K inhibitors type	Control type	Study (year)	Sample size	Receptor type	Population
Alpelisib	Fulvestrant	Rugo et al., 2020 [19]	I(T): 284; C(T): 287	HR-positive, HER2-negative	Postmenopausal women
		André et al., 2019 [20]	I(M): 169; C(M): 172; I(W): 115; C(W): 116	HR-positive, HER2-negative	Men and postmenopausal women

Buparlisib	Fulvestrant	Di Leo et al., 2018 [22]	I(T): 289; C(T): 143	HR-positive, HER2-negative	Postmenopausal women
		Baselga et al.,2017 [23]	I(T): 576; C(T): 571	HR-positive, HER2-negative	Postmenopausal women
	Trastuzumab+paclitaxel	Loibl et al., 2017 [24]	I(T): 25; C(T): 25	HER2-positive	Premenopausal and postmenopausal women
	Paclitaxel	Martín et al., 2016 [26]	I(T): 207; C(T): 209	HER2-negative	Premenopausal and postmenopausal women

Taselisib	Fulvestrant	Saura et al., 2019 [21]	I(M): 73; C(M): 79; I(W): 92; C(W): 89	ER-positive, HER2-negative	Postmenopausal women

Pictilisib	Fulvestrant	Krop et al., ,2016 [25]	I(T): 89; C(T): 79	ER-positive	Postmenopausal women
	Paclitaxel	Vuylsteke et al., 2016 [27]	I(T): 91; C(T): 92	HR-positive, HER2-negative	Premenopausal and postmenopausal women

I: intervention group; C: control group; M: PIK3CA mutated, W: PIK3CA wild-type; T: PIK3CA mutated and wild-type.

**Table 2:** Efficacy of included RCTs of PI3K inhibitors in treatment of breast cancer.

Outcomes	PIK3CA mutation status	Included RCTs	OR (95% CI)	*p*, *I*^2^	Meta-regression	Publication bias	Certainty of evidence
Objective response rate (ORR)	Overall	7 [20, 21-24, 26-27]	1.539 (1.074, 2.204)^∗^	0.015, 54.6%^&^		0.683, 0.000	Low
	Mutated	5 [20-21, 23-24, 26]	1.952 (1.012, 3.766)^∗^	0.030, 62.6%^&^	0.306	0.624, 0.715	Moderate
	Alpelisib	1[20]	2.474 (1.410, 4.343)^∗^		0.785		
	Buparlisib	3 [23-24, 26]	1.481 (0.270, 8.112)	0.009, 78.6%^&^		1.000, 0.996	Moderate
	Taselisib	1 [21]	2.093 (1.094, 4.002)^∗^				
	Wild-type	3 [21, 23-24]	1.252 (0.833, 1.882)	0.630, 0.0%		0.602, 0.251	Moderate
	Total	3 [22, 26-27]	1.303 (0.621, 2.734)	0.075, 61.3%^&^		0.117, 0.029	Low

Progression-free survival (PFS)							
6m-PFS	Overall	7 [19-20, 22-23, 25-27]	1.189 (0.965, 1.465)	0.080, 37.1%	0.292	0.152, 0.000	Moderate
	Mutated	6 [20, 22-23, 25-27]	1.519 (1.144, 2.018)^∗^	0.841, 0.0%		0.188, 0.761	High
	Alpelisib	1 [20]	1.549 (1.010, 2.376)^∗^		0.678		
	Buparlisib	3 [22, 23, 26]	1.427 (0.924, 2.205)	0.396, 0.0%		0.602, 0.859	Moderate
	Pictilisib	2 [25, 27]	1.736 (0.802, 3.761)	0.935, 0.0%		0.371, -	Moderate
	Wild-type	3 [20, 22, 26]	0.938 (0.575, 1.529)	0.132, 50.7%^&^		0.117, 0.106	Low
	Total	4 [19, 23, 25, 27]	1.134 (0.806, 1.596)	0.088, 50.6%^&^		1.000, 0.354	Moderate

1y-PFS	Overall	6 [19, 20, 22-23, 25-26]	1.188 (0.880, 1.603)	0.095, 36.9%	0.840	0.353, 0.000	Moderate
	Mutated	5 [20, 22-23, 25-26]	1.392 (0.972, 1.992)	0.471, 0.0%		1.000, 0.686	High
	Wild-type	3 [20, 22, 26]	0.931 (0.278, 3.115)	0.163, 44.8%		0.117, 0.084	Moderate
	Total	3 [19, 23, 25]	1.210 (0.756, 1.938)	0.048, 62.0%^&^		0.497, 0.384	Low

1.5y-PFS	Overall	6 [19, 20, 23-24, 26-27]	1.506 (1.071, 2.119)^∗^	0.892, 0.0%	0.725	0.173, 0.021	Moderate
	Mutated	5 [20, 23-24, 26-27]	1.577 (0.907, 2.740)	0.865, 0.0%		0.624, 0.693	High
	Wild-type	2 [23, 26]	1.995 (0.632, 6.294)	0.755, 0.0%		0.317, -	Moderate
	Total	3 [19, 24, 27]	1.368 (0.829, 2.258)	0.354, 7.8%		0.042, 0.079	Moderate

2y-PFS	Overall	4 [19-20, 24, 26]	1.716 (0.758, 3.885)	0.934, 0.0%	0.986	0.564, -	High
	Mutated	2 [20, 26]	1.840 (0.491, 6.898)	0.818, 0.0%		0.317, -	Moderate
	Wild-type	2 [24, 26]	1.360 (0.219, 0.435)	0.733, 0.0%		0.317, -	Moderate
	Total	1 [19]	1.771 (0.475, 6.603)	0.303, 5.8%		0.317, -	Moderate

PFS from HR data	Overall	7 [19-20, 22-23, 25-27]	-0.271 (-0.369, -0.173)^∗^	0.342, 10.3%	0.315	0.126, 0.000	Moderate
	Mutated	7 [19-20, 22-23, 25-27]	-0.346 (-0.525, -0.168)^∗^	0.235, 25.4%		0.293, 0.586	High
	Alpelisib	2 [19-20]	-0.431 (-0.658, -0.203)^∗^	1.000, 0.0%	0.398	0.371, -	
	Buparlisib	3 [22, 23, 26]	-0.324 (-0.526, -0.123)^∗^	0.046, 67.6%^&^		0.602, 0.602	Moderate
	Pictilisib	2 [25, 27]	-0.171 (-0.607, 0.266)	0.415, 0.0%		0.371, -	
	Wild-type	3 [20, 22, 26]	-0.168 (-0.413, 0.077)	0.306, 15.5%		0.117, 0.003	Low
	Total	3 [23, 25, 27]	-0.238 (-0.364, -0.112)^∗^	0.647, 0.0%		0.602, 0.678	Low

∗Significant differences, ^&^Substantial heterogeneity.

**Table 3:** Adverse events of included RCTs of PI3K inhibitors in treatment of breast cancer.

System	Outcomes	PI3K inhibitor type	Included RCTs	OR (95% CI)	*p*, *I*^2^	Meta-regression	Publication bias	Certainty of evidence
Gastrointestinal disorders	Diarrhoea (all AE)	Overall	6 [19, 21-24, 26]	3.310 (2.211, 4.955)^∗^	0.001, 75.6%^&^	0.015^#^	0.092, 1.000	Low
		Alpelisib	1 [19]	7.350 (4.947, 10.919)^∗^	—			
		Buparlisib	4 [22-24, 28]	2.710 (2.182, 3.366)^∗^	0.738, 0.0%			
		Taselisib	1 [21]	2.786 (1.546, 5.021)^∗^	—			
	Diarrhoea (3-5 AE)	Overall	7 [19, 21-26]	2.438 (1.404, 4.231)^∗^	0.293, 17.9%	0.113	0.230, 0.165	High
		Alpelisib	1 [19]	1.300 (0.647, 2.612)				
		Buparlisib	4 [22-24, 26]	2.868 (1.507, 5.459)^∗^	0.790, 0.0%			
		Taselisib	1 [21]	8.352 (1.033, 67.540)^∗^				
		Pictilisib	1 [25]	14.455 (0.812, 257.300)				
	Nausea (all AE)	Overall	6 [19, 21-24, 26]	2.241 (1.896, 2.649) ^∗^	0.930, 0.0%	0.277	1.000, 0.726	High
		Alpelisib	1 [19]	2.819 (1.960, 4.053)^∗^				
		Buparlisib	4 [22-24, 26]	2.120 (1.739, 2.585)^∗^	0.930, 0.0%			
		Taselisib	1 [21]	1.991 (1.084, 3.659^∗^				High
	Nausea (3-5 AE)	Overall	6 [19, 21-23, 25-26]	1.405 (0.604, 3.269)	0.288, 19.3%	0.146	0.707, 0.489	
		Alpelisib	1 [19]	7.227 (0.883, 59.126)				
		Buparlisib	3 [22-23, 26]	0.915 (0.425, 1.972)	0.527, 0.0%			
		Taselisib	1 [21]	3.018 (0.122, 74.618)				
		Pictilisib	1 [25]	6.434 (0.327, 126.509)				
	Vomiting (all AE)	Overall	4 [19, 23-24, 26]	1.739 (0.943, 3.206)	0.003,78.8%^&^	0.066	0.734, 0.767	Moderate
		Alpelisib	1 [19]	3.441 (2.151, 5.503)^∗^	—			
		Buparlisib	3 [23-24, 26]	1.228 (0.929, 1.624)	0.791, 0.0%			
	Vomiting (3-5 AE)	Overall	4 [19, 23, 25-26]	1.636 (0.789, 3.392)	0.897, 0.0%	0.657	0.734, 0.811	High
		Alpelisib	1 [19]	2.028 (0.183, 22.496)				
		Buparlisib	2 [23, 26]	1.497 (0.665, 3.372)	0.565, 0.0%			
		Pictilisib	1 [25]	2.721 (0.277, 26.704)				
	Decreased appetite (all AE)	Overall	4 [19, 22-23, 26]	3.541 (2.731, 4.590)^∗^	0.349, 8.7%	0.280	0.308, 0.456	High
		Alpelisib	1 [19]	4.728 (3.016, 7.411)^∗^				
		Buparlisib	3 [22-23, 26]	3.180 (2.403, 4.208)^∗^	0.568, 0.0%			
	Decreased appetite (3-5 AE)	Overall	4 [19, 22-23, 26]	3.207 (1.044, 9.853)^∗^	0.652, 0.0%	0.714	0.308, 0.144	High
		Alpelisib	1 [19]	2.028 (0.183, 22.496)				
		Buparlisib	3 [22-23, 26]	3.643 (1.024, 12.958)^∗^	0.486, 0.0%			
	Stomatitis (all AE)	Overall	6 [19, 21-24, 26]	3.741 (2.924, 4.785)^∗^	0.696, 0.0%	0.333	1.000, 0.735	High
		Alpelisib	1 [19]	4.888 (2.825, 8.458)^∗^				
		Buparlisib	4 [22-24, 26]	3.415 (2.564, 4.548)^∗^	0.710, 0.0%			
		Taselisib	1 [21]	4.660 (1.713, 12.67)^∗^				
	Stomatitis (3-5 AE)	Overall	6 [19, 21-24, 26]	4.482 (1.767, 11.371)	0.967, 0.0%	0.834	0.452, 0.081	Moderate
		Alpelisib	1 [19]	15.541 (0.883, 273.393)				
		Buparlisib	4 [22-24, 26]	3.973 (1.412, 11.175)^∗^	0.994, 0.0%			
		Taselisib	1 [21]	3.018 (0.122, 74.618)				

General and skin disorders	Fatigue (all AE)	Overall	6 [19, 21-24, 26]	1.234 (1.018, 1.496)^∗^	0.318, 14.9%	0.385	0.260, 0.097	Moderate
		Alpelisib	1 [19]	1.559 (1.035, 2.349)^∗^				
		Buparlisib	4 [22-24, 26]	1.272 (1.041, 1.554)^∗^	0.655, 0.0%			
		Taselisib	1 [21]	0.782 (0.464, 1.316)				
	Fatigue (3-5 AE)	Overall	7 [19, 22-26]	2.791 (1.689, 4.613)^∗^	0.608, 0.0%	0.341	0.707, 0.805	High
		Alpelisib	1 [19]	3.455 (0.941, 12.688)				
		Buparlisib	4 [22-24, 26]	2.526 (1.451, 4.398)^∗^	0.553, 0.0%			
		Pictilisib	1 [25]	14.455 (0.812, 257.300)				
	Rash (all AE)	Overall	6 [19, 21-24, 26]	4.403 (2.587, 7.492)^∗^	0.002, 74.2%^&^	0.953	0.707, 0.522	Moderate
		Alpelisib	1 [19]	8.766 (5.072, 15.148) ^∗^				
		Buparlisib	4 [22-24, 26]	3.902 (1.964, 7.753)^∗^	0.004, 77.7%^&^			
		Taselisib	1 [21]	2.735 (0.953, 7.853)				
	Rash (3-5 AE)	Overall	6 [19, 21-24, 26]	14.634 (6.090, 35.160)^∗^	0.688, 0.0%	0.955	1.000, 0.645	High
		Alpelisib	1 [19]	31.281 (4.226, 231.549)				
		Buparlisib	4 [22-24, 26]	13.499 (4.204,43.349)^∗^	0.362, 6.2%			
		Taselisib	1 [21]	7.128 (0.365, 139.069)				

Metabolism and nervous system disorders	Hyperglycaemia (all AE)	Overall	4 [19, 21-22, 24]	7.720 (3.035, 19.637)^∗^	0.000, 88.4%^&^	0.406	0.734, 0.767	Moderate
		Alpelisib	1 [19]	16.255 (10.273, 25.719)^∗^				
		Buparlisib	2 [22, 26]	9.603 (2.823, 32.666)^∗^	0.032, 78.3%^&^			
		Taselisib	1 [21]	2.168 (1.045, 4.495)^∗^				
	Hyperglycaemia (3-5 AE)	Overall	5 [19, 21-22, 25-26]	30.844 (11.114, 85.602)^∗^	0.359, 8.3%	0.474	0.086, 0.064	Moderate
		Alpelisib	1 [19]	82.333 (20.070, 337.758)^∗^				
		Buparlisib	2 [22, 26]	24.869 (4.825, 128.190)^∗^	0.680, 0.0%			
		Taselisib	1 [21]	5.060 (0.241, 106.208)				
		Pictilisib	1 [25]	8.368 (0.443, 157.925)				
	Headache (all AE)	Overall	4 [21-23, 26]	0.981 (0.768, 1.252)	0.842, 0.0%	0.774	0.308, 0.053	High
		Buparlisib	1 [21]	0.995 (0.768, 1.291)	0.681, 0.0%			
		Taselisib	3 [22, 23, 26]	0.877 (0.431, 1.785)	0.831, 0.0%			
	Headache (3-5 AE)	Overall	4 [21-23, 26]	0.838 (0.522, 1.346)	0.842, 0.0%	0.881	0.734, 0.665	High
		Buparlisib	1 [21]	0.808 (0.427, 1.527)	0.669, 0.0%			
		Taselisib	3 [22-23, 26]	0.877 (0.431,1.785)				

^∗^Significant differences, ^&^Substantial heterogeneity, ^#^Source of heterogeneity.

## Supplementary Materials

Supplementary 1 Table S1 Search strategies.

Supplementary 2 Table S2 Baseline characteristic of included nine studies;

Supplementary 3 Figure S1 Risk of bias summary.

Supplementary 4 Figure S2 Network funnel plot for objective response rate.

Supplementary 5 Figure S3 Sensitivity analysis for objective response rate (A) and 6-month progression-free survival (B).
