# Supplementary material for: Which Is the Most Appropriate PI3K Inhibitor for Breast Cancer Patients with or without PIK3CA Status Mutant? A Systematic Review and Network Meta-Analysis
Source: Biomed Res Int. 2020 Dec 3;2020:7451576. doi: 10.1155/2020/7451576 (PMC7739049; doi:10.1155/2020/7451576)
Supplement: Supplementary 1 — Table S1 Search strategies. [file 7451576.f1.doc]

**Supplementary Table 1 Search strategies**

**Search strategies for PubMed**

#1. Search: (PIK3CA mutate) AND (breast cancer)

#2. Search: PI3K inhibitor

#3. #1 and #2

**Search strategies for EMbase**

#1. pik3ca AND mutate AND pi3k AND inhibitor AND breast AND cancer

#2.pik3ca AND mutate AND breast AND cancer

#3. #1 and #2

**Search strategies for Cochrane library**

#1.PIK3CA mutate breast cancer
