# Supplementary material for: Which Is the Most Appropriate PI3K Inhibitor for Breast Cancer Patients with or without PIK3CA Status Mutant? A Systematic Review and Network Meta-Analysis
Source: Biomed Res Int. 2020 Dec 3;2020:7451576. doi: 10.1155/2020/7451576 (PMC7739049; doi:10.1155/2020/7451576)
Supplement: Supplementary 2 — Table S2 Baseline characteristic of included nine studies; [file 7451576.f2.doc]

**Table S2 Baseline characteristic of included nine studies.**

| **Study(year)** | **Study type** | **Sample size(gender)** | **Age** | **Cancer type** | **Population** | **ECOG score(0/1)** | **Stage** | **PI3K inhibitor regimen** | **Intervention arm** | **Control arm** | **Period** |
| --- | --- | --- | --- | --- | --- | --- | --- | --- | --- | --- | --- |
| Rugo HS,2020[1] | RCT, Phase 3 | I(T):284  C(T):287 | - | HR-Positive, HER2-negative Advanced BC | Postmenopausal women | - | - | Alpelisib, mean 248 mg/day | Alpelisib+ Fulvestrant | Placebo+ Fulvestrant | During the on-treatment period |
| André F,2019[2] | RCT, Phase 3 | I(M):169(1/168)  C(M):172(0/172)  I(W):115(0/115)  C(W):116(0/116) | I(M):63(25–87)  C(M):64(38–92)  I(W):62(39–82)  C(W):63(32–88) | HR-Positive, HER2-negative Advanced BC | Men and postmenopausal women | I:(M)122/56  C(M):113/58  I(W):84/30  C(W):79/37 |  | Alpelisib(300 mg, at beginning) | Alpelisib+ Fulvestrant | Placebo+ Fulvestrant | every 28 days |
| Saura C,2019[3] | RCT, Phase 2 | I(M):73  C(M):79  I(W):92  C(W):89 | I(M):64.8±9.1  C(M):63.6 ±9.0  I(W):64.5 ±8.1  C(W):65.6 ±8.4 | ER-Positive, HER2-negative early-stage BC | Postmenopausal women | I(M):65/8  C(M):70/9  I(W):81/10  C(W):76/13 | I(M):68/5  C(M):71/8  I(W):82/10  C(W):80/9 | Taselisib, (4 mg) | Taselisib+ Fulvestrant | Fulvestrant | on a 5 days-on, 2 days-off schedule) for 16 weeks |
| Di Leo A,2018[4] | RCT, Phase 3 | I(T):289  C(T):143 | I(T):60.0 (54.0–68.0)  C(T):62.0 (55.0–69.0) | HR-Positive, HER2-negative Advanced BC | Postmenopausal women | I(T):173/112  C(T):91/48 |  | Buparlisib (100 mg) | Buparlisib+ Fulvestrant | Placebo+ Fulvestrant | Days 1 and 15 of cycle 1 and on day 1 of subsequent 28-day cycles |
| Baselga J,2017[5] | RCT, Phase 3 | I(T):576  C(T):571 | I(T):62 (55–69)  C(T):61 (54–68) | HR-Positive, HER2-negative Advanced BC | Postmenopausal women | I(T):333/231  C(T):344/211 |  | Buparlisib,  100 mg/day | Buparlisib+ Fulvestrant | Placebo+ Fulvestrant | Days 1 and 15 of cycle 1, and on day 1 of subsequent 28-day cycles |
| Loibl, S,2017[6] | RCT, Phase 2 | I(T):25  C(T):25 | I(T):50.0 (35.0-72.0)  C(T):50.0 (26.0e78.0) | HER2+ primary breast cancer | Premenopausal and postmenopausal women |  | I(T):24/1  C(T):25/0 | Buparlisib, 4 mg/kg | Buparlisib + trastuzumab + paclitaxel | Placebo + trastuzumab + paclitaxel | Overall 18-week treatment |
| Krop, I. E,2016[7] | RCT, Phase 2 | I(T):89  C(T):79 | I(T):60 (36–90)  C(T):63 (40–82) | ER-Positive, Advanced BC | Postmenopausal women |  |  | Pictilisib, 340 mg | Pictilisib + Fulvestrant | Placebo+ Fulvestrant | day 1 and day 15 of cycle 1 and day 1 of subsequent cycles in both groups |
| Martín M, 2016[8] | RCT, Phase 2 | I(T):207  C(T):209 | I(T):55 (25-84)  C(T):56 (24-78) | HER2-negative, advanced/metastatic BC | Premenopausal and postmenopausal women | I(T):135/69  C(T):146/60 |  | Buparlisib,  100 mg/day | Buparlisib +  paclitaxel | Placebo +  paclitaxel | 28-day treatment cycles |
| Vuylsteke P, 2016[9] | RCT, Phase 2 | I(T):91  C(T):92 | I(T):55.0 (30–78)  C(T):58.0 (33–80) | HR-Positive, HER2-negative Advanced BC | Premenopausal and postmenopausal women | I(T):52/37  C(T):43/49 |  | Pictilisib, 260 mg | Pictilisib + paclitaxel | Placebo + paclitaxel | 30-day treatment cycles |
