# Supplementary material for: Which Is the Most Appropriate PI3K Inhibitor for Breast Cancer Patients with or without PIK3CA Status Mutant? A Systematic Review and Network Meta-Analysis
Source: Biomed Res Int. 2020 Dec 3;2020:7451576. doi: 10.1155/2020/7451576 (PMC7739049; doi:10.1155/2020/7451576)
Supplement: Supplementary 3 — Figure S1 Risk of bias summary. [file 7451576.f3.pdf]

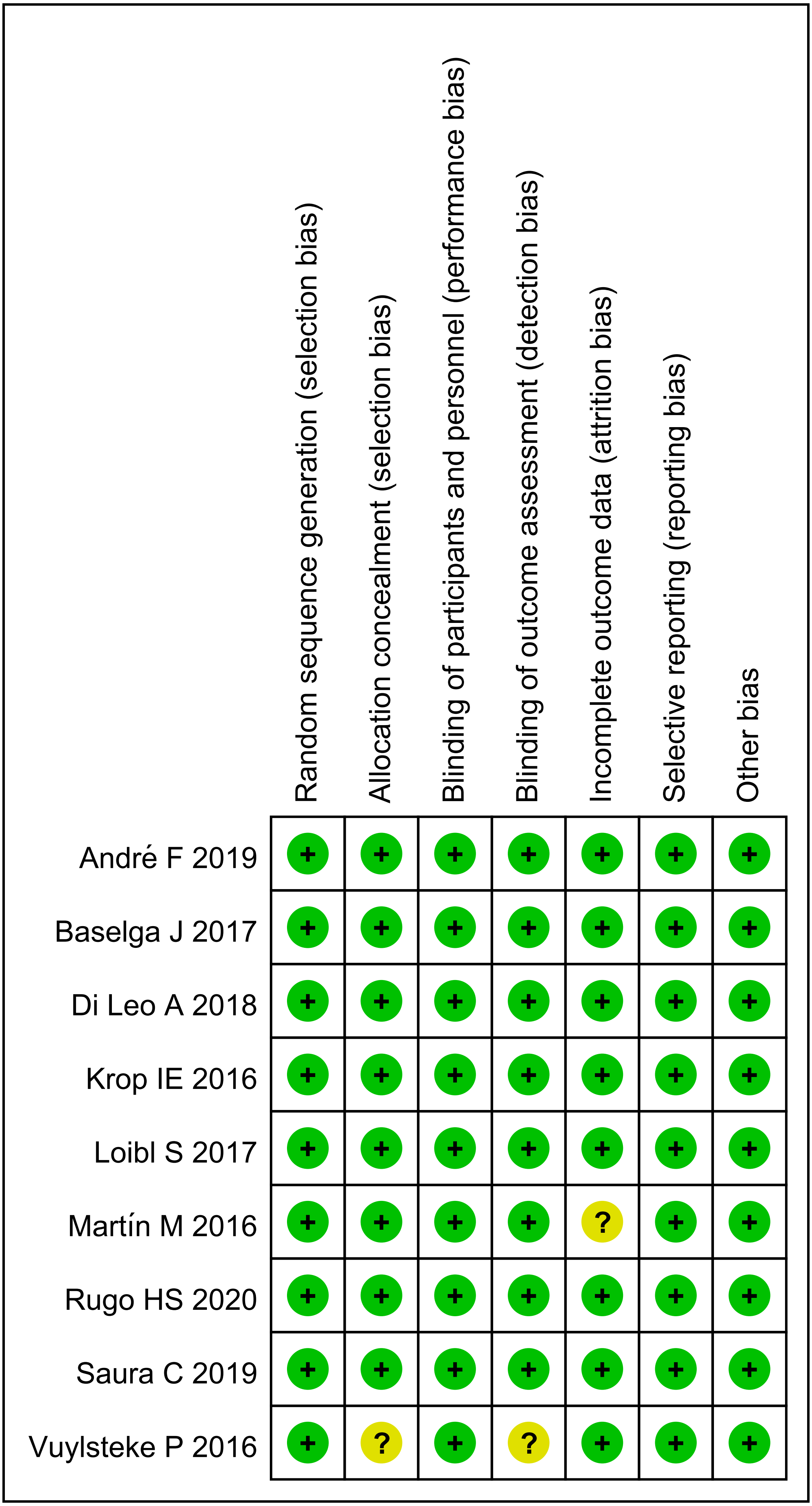
Random sequence generation (selection bias)
Allocation concealment (selection bias)
Blinding of participants and personnel (performance bias)
Blinding of outcome assessment (detection bias)
Incomplete outcome data (attrition bias)
Selective reporting (reporting bias)
Other bias
André F 2019
Baselga J 2017
Di Leo A 2018
Krop IE 2016
Loibl S 2017
Martín M 2016
Rugo HS 2020
Saura C 2019
Vuylsteke P 2016
