# Supplementary material for: Which Is the Most Appropriate PI3K Inhibitor for Breast Cancer Patients with or without PIK3CA Status Mutant? A Systematic Review and Network Meta-Analysis
Source: Biomed Res Int. 2020 Dec 3;2020:7451576. doi: 10.1155/2020/7451576 (PMC7739049; doi:10.1155/2020/7451576)
Supplement: Supplementary 4 — Figure S2 Network funnel plot for objective response rate. [file 7451576.f4.pdf]

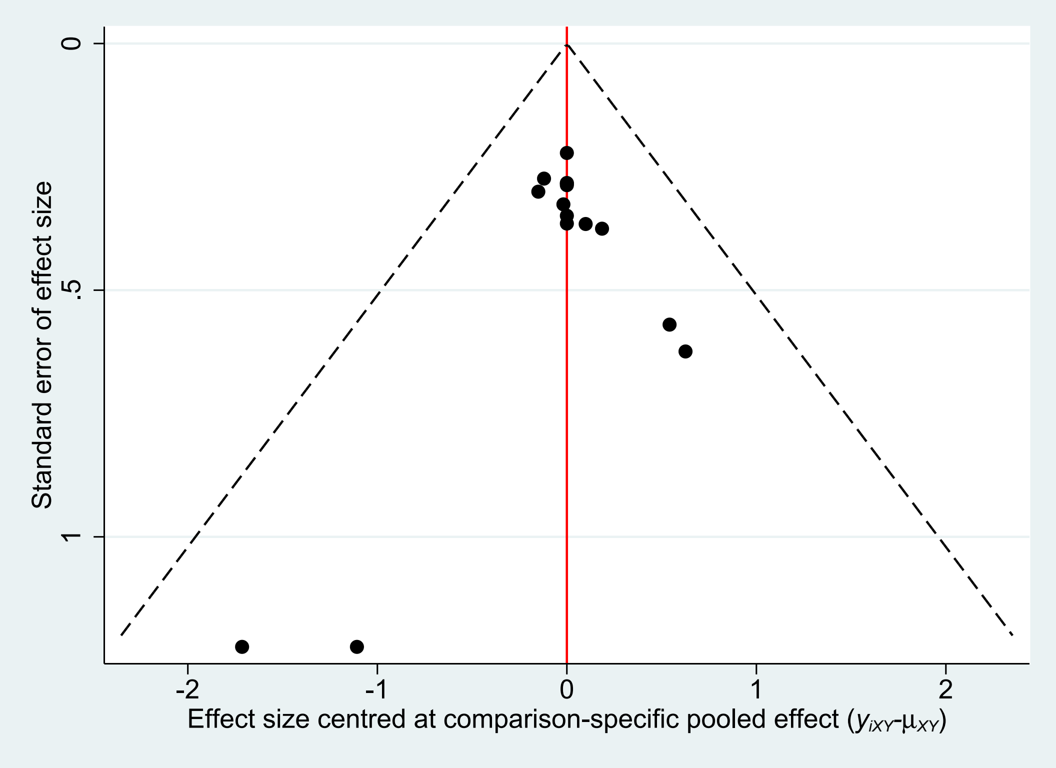
0
.5
1
Standard error of effect size
-2
-1
0
1
2
Effect size centred at comparison-specific pooled effect (y_{iXY}-μ_{XY})
