# Supplementary figures and images for: Which Is the Most Appropriate PI3K Inhibitor for Breast Cancer Patients with or without PIK3CA Status Mutant? A Systematic Review and Network Meta-Analysis
Source: Biomed Res Int. 2020 Dec 3;2020:7451576. doi: 10.1155/2020/7451576 (PMC7739049; doi:10.1155/2020/7451576)

A

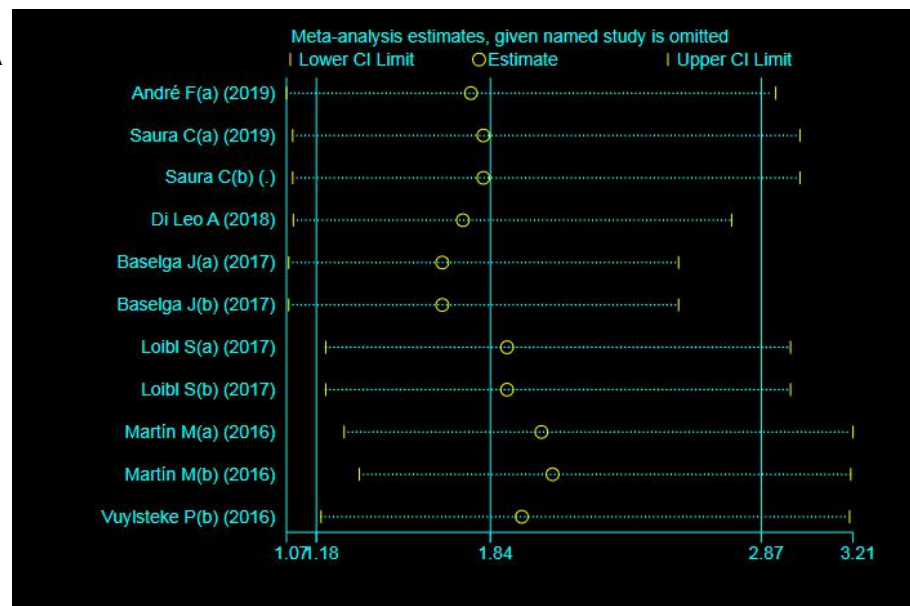

B

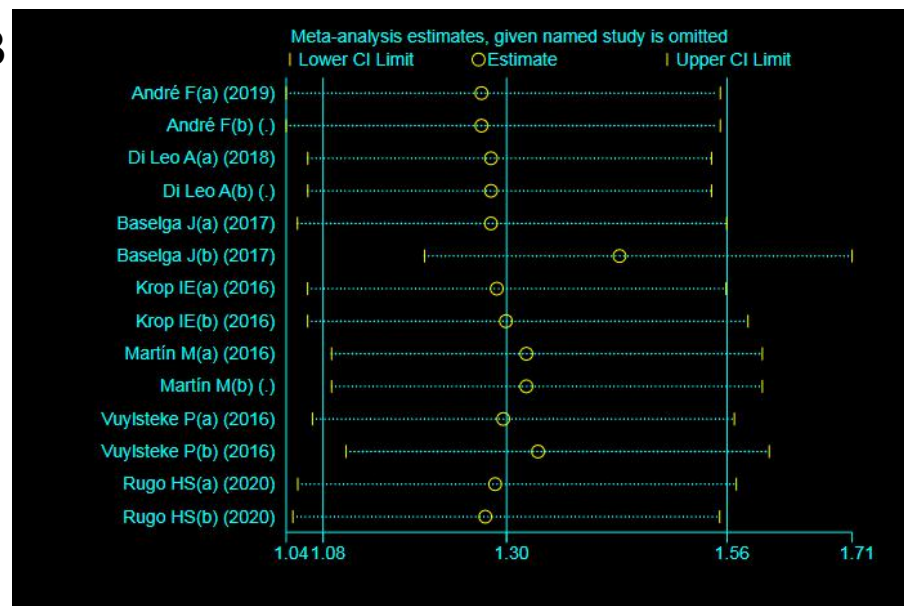

Supplement: Supplementary 5 — Figure S3 Sensitivity analysis for objective response rate (A) and 6-month progression-free survival (B). [file 7451576.f5.pdf]
